# Beneficial aspects of real time flow measurements for the management of acute right ventricular heart failure following continuous flow ventricular assist device implantation

**DOI:** 10.1186/1749-8090-7-119

**Published:** 2012-11-12

**Authors:** Sotirios Spiliopoulos, Dilek Guersoy, Reiner Koerfer, Gero Tenderich

**Affiliations:** 1 Department for the Surgical Management of End-stage Heart Failure and Mechanical Circulatory Support, Heart and Vascular Center Duisburg, Fahrner street 133-135, 47169, Duisburg, Germany

**Keywords:** Heart failure, Right ventricle, Ventricular assist devices

## Abstract

**Background:**

Optimal management of acute right heart failure following the implantation of a left ventricular assist device requires a reliable estimation of left ventricular preload and contractility. This is possible by real-time pump blood flow measurements.

**Clinical case:**

We performed implantation of a continuous flow left ventricular assist device in a 66 years old female patient with an end-stage heart failure on the grounds of a dilated cardiomyopathy. Real-time pump blood flow was directly measured by an ultrasonic flow probe placed around the outflow graft.

**Diagnosis:**

The progressive decline of real time flow and the loss of pulsatility were associated with an increase of central venous pressure, inotropic therapy and progressive renal failure suggesting the presence of an acute right heart failure. Diagnosis was validated by echocardiography and thermodilution measurements.

**Treatment:**

Temporary mechanical circulatory support of the right ventricle was successfully performed. Real time flow measurement proved to be a useful tool for the diagnosis and ultimately for the management of right heart failure including the weaning from extracorporeal membrane oxygenation.

## Background

Right heart failure is a common complication following left ventricular circulatory support [1]. Diagnosis and management are usually based upon echocardiographic findings and measurements of mixed venous saturation which provide information about left ventricular preload and left ventricular contractility. Alternatively this information could be easily obtained by the pump blood flow estimated by the left ventricular assist device. However this would not be accurate since currently available assist devices do not measure real-time blood flow but only calculate it using the rotational speed of the pump and the power consumption of the motor. Furthermore this calculation would be susceptible to external factors such as blood viscosity [2]. In contrast, real-time blood flow measurements are accurate. In this paper we describe a case of an acute right heart failure following left ventricular circulatory support which was diagnosed and successfully managed based upon information obtained through real-time blood flow measurements.

## Case presentation

A continuous flow left ventricular assist device (HeartAssist 5; Micromed Cardiovascular Inc. Houston, USA) was implanted in a 66 years old female patient with an end-stage heart failure NYHA IV with a history of dilated cardiomyopathy. Preoperative echocardiography showed a severe impairment of the left ventricular function (EF 10%), a secondary pulmonary hypertension with an estimated systolic pulmonary artery pressure of 48 mmHg and a right ventricular dysfunction with a tricuspid annular plane systolic excursion (TAPSE) of 15 mm. The cardiac output, determined by right heart catheterization, was 3,5 L/min (Cardiac Index 1,92 L/min/m^2^) with a systemic peripheral vascular resistance of 1350 dyn.sec.cm5 and pulmonary vascular resistance of 383 dyn·s/cm5). After four days intravenous therapy with milrinone (14 μg/kg/hr)., cardiac output improved to 4,6 L/min (Cardiac Index 2,5 L/min/m^2^) with a systemic vascular resistance of 1000 dyn·s/cm5 and pulmonary vascular resistance decreased to 190 dyn·s/ cm5. After implant of the HeartAssist5, the thorax could not be completely closed, due to severe fluid retention and oedema. Complete closure resulted in severe right ventricular failure. During the first and the second postoperative day the mean real-time flow was 4,8 L/min (range: 4,4 L/min-5,2 L/min) at a fixed pump speed of 8400 rpm (range: 8300–8500 rpm) and the flow waveforms showed very pulsatile flows (2.0 L/min in diastole and 6.5 L/min in systole). In the third postoperative day the mean real-time flow decreased to 4,3 L/min (range: 3,9-5,1 L/min) and the flow curve became flat. Real time flow was correlated to cardiac output measured by thermodilution (4,07 L/min on a systemic peripheral vascular resistance of 766 dyn·s/cm5). Initialy a low-dose inotropic therapy with milrinone (3 μg/kg/hr), noradrenalin (1 μg/kg/hr) and dopamine (200 μg/kg/hr) was enough to restore cardiac output (4,7 L/min with a systemic vascular resistance of 938 dyn·s/cm5). Unfortunately, in the eighth postoperative day, the central venous pressure increased from 9 mmHg to a mean 16 mmHg (range 14–20 mmHg) and the real-time flow decreased (mean 3,9 L/min, range 3,5-4,7 L/min). 24 hours later the patient developed renal failure, necessitating haemodialysis and a high dose inotropic therapy became mandatory (norepinephrine 8 μg/kg/hr, Milrinone 11 μg/kg/hr). Despite maximal inotropic therapy, flows continued to decline and on the tenth postoperative day the patient was placed on venoarterial extracorporeal membrane oxygenation (Levitronix Centrimag, Zurich, Switzerland). The two-stage venous cannula was inserted to the right atrium and the arterial cannula in the main pulmonary artery. Real time flow & pulsatility recovered promptly. Mean flow values over the first 24 hours ranged from 4,8 L/min to 6,2 L/min at a fixed pump speed of 8700 rpm. After four days of ECMO, the inotropes were weaned. At the sixth day of ECMO support, weaning from ECMO was started. Mean real time flow remained stable over the weaning procedure (5 L/min; range 4,3-5,5 L/min). The ECMO circuit was disconnected at the 14^th^ post-operative day. Three days after ECMO removal, the Thorax was closed, and the further course was uneventful.

## Conclusions

Currently available continuous flow ventricular assist devices estimate the pump blood flow with a calculation using the power consumption of the motor and the rotational speed of the pump [3]. However, it is known that the estimated blood flow depends to a large extent from external factors such as blood viscosity [2], rotational speed and loading of the ventricle. The estimated blood flow is therefore not accurate. Right heart failure is a common complication following left ventricular circulatory support [1]. The main reasons are a shift of the interventricular septum to the left and the inability of a dysfunctional right ventricle to cope with the increased venous return, delivered by the LVAD. It is therefore essential for the management of right ventricular failure to precisely determine the left ventricular preload and contractility. This is quite well possible by the means of transesophageal echocardiography and mixed venous saturation but is only available in the early post-op phase. The HeartAssist 5 left ventricular assist device instead has an ultrasonic flow probe placed around the outflow graft which provides real-time flow measurement [4]. This measurement is reliable and gives information about the left ventricular preload and the left ventricular contractility [5]. In our case real time flow was repeatedly validated by haemodynamic measurements obtained by thermodilution. The progressive decline of real time flow and loss of pulsatility was associated with an increase of central venous pressure, inotropic therapy and progressive renal failure requiring ultimately a mechanical circulatory support of the right ventricle. Furthermore the real time flow measurement proved to be a useful tool for the weaning from the extracorporeal membrane oxygenation since it enabled the monitoring of the left ventricular preload & contractility (0.6 – 8.3 L/min) throughout the post-operative course. The loss of pulsatility in the flow curve is an early warning sign for insufficient preload of the LV, which will allow earlier intervention and therefore may help to better manage critically ill patients. Furthermore real-time flow measurements proved to be a useful tool and a reliable alternative to conventional techniques for the measurement of cardiac output in the clinical setting.

## Consent

Written informed consent was obtained from the patient for publication of this report.

## Competing interest

The authors exclude any conflict of interest.

## Authors’ contributions

SS drafted the manuscript. DG critically reviewed the manuscript. RK and GT approved the manuscript for submission. All authors read and approved the final manuscript.
